# Detecting neurodegenerative disorders from web search signals

**DOI:** 10.1038/s41746-018-0016-6

**Published:** 2018-04-23

**Authors:** Ryen W. White, P. Murali Doraiswamy, Eric Horvitz

**Affiliations:** 1 Microsoft, Bellevue, WA 98004 USA; 2 Duke University Health System and Duke Institute for Brain Sciences, Durham, NC 27710 USA; 3 Microsoft, Redmond, WA 98052 USA

**Keywords:** Neurological disorders, Public health

## Abstract

Neurodegenerative disorders, such as Parkinson’s disease (PD) and Alzheimer’s disease (AD), are important public health problems warranting early detection. We trained machine-learned classifiers on the longitudinal search logs of 31,321,773 search engine users to automatically detect neurodegenerative disorders. Several digital phenotypes with high discriminatory weights for detecting these disorders are identified. Classifier sensitivities for PD detection are 94.2/83.1/42.0/34.6% at false positive rates (FPRs) of 20/10/1/0.1%, respectively. Preliminary analysis shows similar performance for AD detection. Subject to further refinement of accuracy and reproducibility, these findings show the promise of web search digital phenotypes as adjunctive screening tools for neurodegenerative disorders.

## Introduction

Neurodegenerative disorders (NDs) are prevalent and a major source of healthcare expenditure.^1^ NDs progress slowly,^2^ and their symptoms may be subtle and mistaken for more common conditions.^3, 4^ Early detection of NDs enables earlier intervention, which can slow their progression. This study examines the use of digital phenotypes^5^ for detecting NDs, operationalized as patterns of search activity gathered during engagement with web search engines. Methods based on these observational data show promise in offering new pathways for the early detection of brain disease.

Prior studies with large-scale logs of the search activity of millions of people have highlighted opportunities for detection of cancer^6, 7^ and for disease surveillance.^8, 9^ This study investigates how analyses of longitudinal log data from search engines might help detect evidence of Parkinson’s disease (PD), a common progressive ND affecting some 7–10 million people worldwide. Dopaminergic deficiency in PD results in symptoms such as tremors and cognitive decline,^10^ evidence of which may be apparent in search log signals. PD is challenging to diagnose: the current accuracy of clinical diagnosis of probable PD for patients presenting with motor symptoms in primary care settings is around 80%, with limited improvements in the past 25 years, especially at early disease stages.^11^ Hence, there is a need for a simple scalable test that can be used for screening in the community or at home. This work also explores whether classifiers using search log signals can help with diagnostic challenges in PD, specifically distinguishing early PD from essential tremor (ET).^3, 4^

This study uses a total of 18 months of deidentified logs of United States search activity from the Microsoft Bing web search engine, comprising millions of English-speaking searchers from September 2015 to February 2017 inclusive. These data are routinely collected for improving search results and permitted through Bing’s Terms of Service. A range of observational features were computed per searcher over the duration of the logs: (1) Symptom: presence of PD symptom-related query terms (including synonyms) derived from published literature; (2) Motor: motor symptoms such as cursor movements, including speed, direction changes, tremors (defined as horizontal or vertical oscillations in cursor position up to 20 pixels in each direction), and vertical scrolling. Cursor position data were sampled while the cursor was in motion; (3) Repetition: presence of repeat queries, repeat result clicks, and repeat query-result click pairs, and (4) Risk Factors: presence of risk factors derived from previous work (e.g.,^12–14^). These included age and gender (inferred using proprietary Bing classifiers), and head trauma, toxin exposure, and familial factors based on terminology appearing in query text. For the *Motor* class, feature values are first computed per query instance and then averaged across all query instances for the searcher. Some features align with criteria used by physicians (e.g., tremors)^10, 15^ while others are more difficult to measure in clinical practice (e.g., memory loss).^16^

From the full set of logs, searchers who input queries containing first-person statements about PD diagnosis (e.g., “just diagnosed with parkinsons”) were identified. These experiential diagnostic queries are used as evidence of receiving a PD diagnosis. Cases exhibiting evidence that diagnostic queries were for others (e.g., father, spouse, etc.) were excluded. Multiple additive regression trees (MART) classifiers^17^ were trained to detect evidence of PD diagnosis from all PD symptom searchers. Advantages of MART include model interpretability, facility for rapid training and testing, and robustness against noisy labels and missing values. There were 703 positive cases, of searchers who queried for symptoms and issued at least one experiential diagnostic query (30.8% of the experiential diagnostic searchers), and 31,321,070 negative cases, of searchers who only issued queries on PD symptoms. The data were used in classifier training as is. The application of sampling methods to correct for class imbalance is left to future work. Since NDs progress slowly^2^ and the observation window is limited to 18 months, the classification task likely identifies the existence of PD rather than forecasting a future diagnosis.

Ten-fold cross validation was used to train and test the classifier. It predicts the input of an experiential diagnostic query for PD with strong performance (area under the receiver operating characteristic curve [AUROC] = 0.9357) using 18 months of search log data. AUROC drops to 0.8626 with 12 months of data, and 0.8151 with 6 months of data. Since false positives can generate unnecessary alarm and additional healthcare utilization in fielded uses (e.g., at population-scale in search engines), low false positive rates (FPRs) are desirable. Classifier sensitivities at FPR = 20/10/1/0.1% are 94.2/83.1/42.0/34.6%, respectively. The results offer evidence that the existence of NDs in searchers is detectable from streams of data from the use of search engines over time. Table 1 shows the list of observational features with non-zero discriminatory weights in the learned classifier. Features related to tremors—both from search terms (e.g., “hands shaking”) and from mouse cursor movements (e.g., estimated rate of cursor position oscillation), repeat queries, and repeat search-result clicks, and the inferred age and gender of searchers, had highest discriminatory weights.

**Table 1:** Features used in PD classifier, ranked by discriminative weight and scored with respect to the top-ranked feature: *TimeBetweenRepeatQueries*. Features are computed over all queries for each searcher. Features from the Motor class are first computed for each query instance and then averaged across all query instances for that searcher

Feature name	Class	Brief description	Weight
TimeBetweenRepeatQueries	Repetition	AVG time between repeat queries	1.000000
FractionOfQueriesAreRepeats	Repetition	% of all queries that are repeat queries	0.971182
NumberOfTremorEvents	Motor	# of tremor events^a^	0.715004
AverageTremorFrequency	Motor	AVG tremor frequency in hertz (# of oscillations/time)	0.595772
FractionOfQueriesHaveSymptoms	Symptom	% of all queries with 1+ symptoms	0.457336
AgeIs50To85	Risk Factors	Inferred searcher age is 50–85 years	0.432355
FractionOfClicksAreRepeats	Repetition	% of result clicks that are repeat clicks on same result	0.341164
FractionOfQueriesHaveRiskFactors	Risk Factors	% of all queries with 1+ risk factors	0.329801
GenderIsFemale	Risk Factors	Inferred gender is female	0.313425
TotalTimeCursorMoving	Motor	Total time mouse cursor is actively moving	0.297699
NumberOfScrollEvents	Motor	# of scroll events	0.259432
NumberOfScrollEventsDownward	Motor	# of scroll events downward	0.256692
AverageScrolVelocity	Motor	AVG scrolling velocity	0.249454
MinimumCursorYCoordinate	Motor	MIN *y*-coordinate of mouse cursor (top of page *y* is 0)	0.247770
FractionOfCursorTransitionsAreDirectionChanges	Motor	% of mouse cursor transitions with direction changes^b^	0.243873
AverageCursorAcceleration	Motor	AVG acceleration of mouse cursor	0.239814
NumberOfHyperlinkClicks	Motor	# of hyperlink clicks	0.239568
AverageCursorVelocity	Motor	AVG velocity of mouse cursor	0.232418
NumberOfCursorTransitionsAreDirectedUpward	Motor	# of transitions directed upward	0.232311
TotalDistanceScrolled	Motor	Total distance scrolled	0.215000
AverageCursorXCoordinate	Motor	AVG *x*-coordinate of mouse cursor (left of page *x* is 0)	0.214955
FractionCursorTimeInWhitespace	Motor	% of time mouse cursor in whitespace^c^	0.211925
MaximumDeviationInPreclickCursorTrail	Motor	MAX deviation in pre-click mouse cursor trail^d^	0.210185
AveragePreclickCursorVelocity	Motor	AVG velocity of mouse cursor before click	0.208572
TotalScrollingTime	Motor	Total time spent scrolling	0.207520
AverageCursorJounce	Motor	AVG jounce of mouse cursor	0.206460
MinimumCursorXCoordinate	Motor	MIN *x*-coordinate of mouse cursor	0.199193
MaximumCursorVelocity	Motor	MAX mouse cursor velocity	0.196639
NumberOfCursorTransitions	Motor	# of mouse cursor transitions between logged points	0.192631
GenderIsMale	Risk Factors	Inferred gender is male	0.191614
AverageCursorVelocity	Motor	AVG velocity of mouse cursor	0.190826
CursorExhibitsReadingBehavior	Motor	Cursor shows evidence of reading behavior^21^	0.190713
FractionCursorMoveTimeHaveTremors	Motor	% of mouse cursor move time having tremor events	0.188127
AverageCursorYCoordinate	Motor	AVG *y*-coordinate of mouse cursor	0.171520
AverageCursorJerk	Motor	AVG jerk of mouse cursor	0.168440
NumberOfTransitionsDirectedRightward	Motor	# of mouse cursor transitions directed rightward	0.157965
TotalNumberOfClicks	Motor	# of mouse clicks, inc. non-hyperlink (in whitespace)	0.153249
AverageAccelerationOfCursor	Motor	AVG acceleration of mouse cursor	0.150714
AgeIs35To49	Risk Factors	Inferred searcher age is 35–49 years	0.145166
NumberOfNonHyperlinkClicks	Motor	# of non-hyperlink mouse clicks	0.132365
MaximumCursorYCoordinate	Motor	MAX *y*-coordinate of mouse cursor	0.127897
NumberOfCursorEvents	Motor	# of mouse cursor events	0.126026
NumberOfScrollEventsUpward	Motor	# of upward scroll events	0.117682
TotalCursorDistanceTraveled	Motor	Total distance traveled by mouse cursor	0.111703
AverageCursorPreclickOverrunDistance	Risk Factors	AVG pre-click mouse cursor link overrun distance^e^	0.110122
AverageCursorPreclickDeviation	Motor	AVG deviation in pre-click mouse cursor trail	0.106043
NumberOfCursorTransitionsDownward	Motor	# of mouse cursor transitions directed downward	0.104645
MaximumCursorJerk	Motor	MAX jerk of mouse cursor	0.098519
NumberOfCursorLoops	Motor	# of 360° loops in mouse cursor movements^f^	0.094700
FractionOfTimeWithCursorInWhitespace	Motor	% of time spent with mouse cursor in whitespace	0.092794
MaximumCursorYCoordinate	Motor	MAX *y*-coordinate of mouse cursor	0.081183
NumberOfCursorTransitionsLeft	Motor	# of mouse cursor transitions directed left	0.074749
MaximumCursorJounce	Motor	MAX jounce of mouse cursor	0.072211
AgeIs25To34	Risk Factors	Inferred searcher age is 25–34 years	0.069420
NumberOfCursorDirectionChanges	Motor	# of mouse cursor direction changes	0.068290
FractionQueriesWithRepeatQueryClick	Repetition	% of queries with repeat query-result click pair	0.045272
MaximumCursorPreclickVelocity	Motor	MAX velocity of pre-click mouse cursor trail	0.035155

^a^ Tremor events are defined as horizontal or vertical oscillations in the position of the mouse cursor, with a mouse cursor movement of no more than 20 pixels in either direction

^b^ Transitions between logged cursor position data points where a change in mouse cursor direction is noted (e.g., moving the mouse cursor leftward then moving the mouse cursor rightward)

^c^ Fraction of total time spent on the search engine result page where the mouse cursor is parked over whitespace (i.e. regions of the result page where there are no elements)

^d^ Average residuals in a line of best fit for the five cursor position data points (i.e. the cursor trail) logged before a hyperlink click

^e^ Total distance traveled (in pixels) by mouse cursor pre-click after initial pass over the target hyperlink

^f^ Number of 360-degree loops in the mouse cursor trails, where a loop is defined as a sequence of direction changes resulting in a circular motion of the mouse cursor (e.g., move right, move down, move left, move up)

**Fig. 1:**
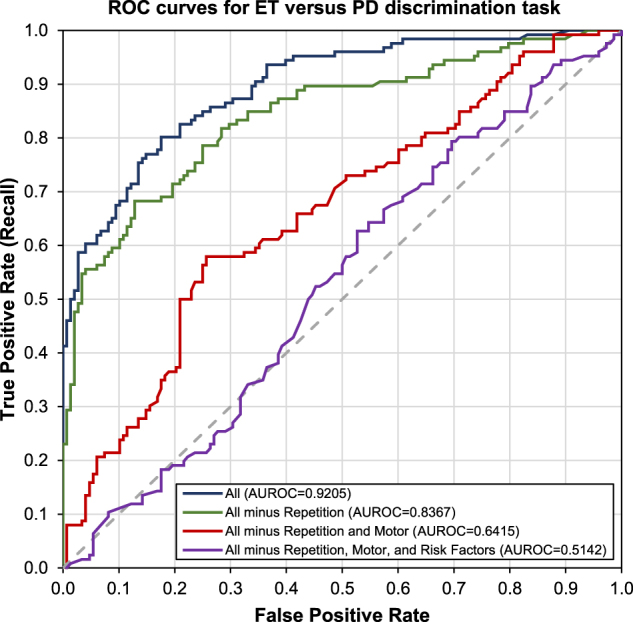
Receiver-operator characteristic curve for the task of discriminating between Parkinson’s disease (PD) and essential tremor (ET), using all features and with feature ablations. Starting with the classifier using all features (All), ablations removed features of the repetition class (all minus repetition), repetition and motor classes (all minus repetition and motor), and repetition, motor, and risk factors classes (all minus repetition, motor, and risk factors). After each class is removed, the classifier is retrained and AUROC is recomputed. When all three classes are removed, the classifier uses only features from the Symptom class (purple line)

Tremors have many explanations, including ET, which shares some symptoms with PD. Distinguishing between ET and early PD is important for tremor sufferers.^3^ Focusing on those who searched for tremors (*n* = 4,262,953), a MART classifier was trained to distinguish PD (*n* = 309) and ET (*n* = 307). Figure 1 shows the ROC curve illustrating strong classifier performance (AUROC = 0.9205) using all features available to the classifier. Features related to scrolling, cursor direction changes, tremor frequencies, and query repetition were important. This is corroborated by ablation studies, where the largest drop in AUROC (23%, *Z* = 7.10, *p* < 0.001^18^) occurs when *Motor* features are excluded. Motor symptoms, including tremor frequencies, are also important in distinguishing ET and PD during clinical examinations.^19^

The classifiers learned from search query and motor interaction data show promise for developing new kinds of diagnostic tools for NDs. The periodic application of these methods may support the study of temporal dynamics in NDs for consenting searchers. They can also help discriminate between illnesses with similar symptoms, as shown with a case study of identifying searchers with experiential diagnostic queries for ET versus PD. The classifier leverages evidence unavailable to physicians (e.g., longitudinal query repetition, mouse cursor activity) that could aid in more traditional clinical diagnoses. Application of these classifiers could help screen for patients with higher ND likelihoods. Surfacing their predictions and confidence scores to physicians could offer additional evidence to help physicians discriminate between conditions. Identifying the specific digital phenotypes (e.g., estimated tremor frequencies) related to NDs that carry most weight for each patient may also have diagnostic utility. It is noted that while experiential diagnostic queries provide evidence of ND, definitive ground truth was unavailable in this study. Future work will expand this analysis to other NDs and perform prospective analyses with clinically diagnosed ND patients at different stages of illness to validate the diagnostic and prognostic utility of digital signals. Preliminary analysis shows that the methods in this study may scale to other NDs, such as Alzheimer’s disease (AUROC = 0.9135, classifier sensitivities at FPR = 20/10/1/0.1% are 91.0/81.5/38.8/26.1%, respectively). A recent study of keystroke typing patterns in verified PD patients^20^ found similar results to those on PD presented herein. The findings of the two studies taken together support the promise of using digital phenotypes for early detection of PD.

### Data availability statement

The data that support the findings of this study are available from Microsoft, but restrictions apply to the availability of these data. Data are however available from the authors upon reasonable request and with permission of Microsoft.
